# The 2023 Impact of Inflammatory Bowel Disease in Canada: Epidemiology of IBD

**DOI:** 10.1093/jcag/gwad004

**Published:** 2023-09-05

**Authors:** Stephanie Coward, Eric I Benchimol, M Ellen Kuenzig, Joseph W Windsor, Charles N Bernstein, Alain Bitton, Jennifer L Jones, Kate Lee, Sanjay K Murthy, Laura E Targownik, Juan-Nicolás Peña-Sánchez, Noelle Rohatinsky, Sara Ghandeharian, James H B Im, Tal Davis, Jake Weinstein, Quinn Goddard, Julia Gorospe, Jennifer Bennett, Léa Caplan, Maxime Bergevin, Xin Yu Yang, Kate Mason, Rhonda Sanderson, Colten Brass, Gilaad G Kaplan

**Affiliations:** Departments of Medicine and Community Health Sciences, University of Calgary, Calgary, Alberta, Canada; SickKids Inflammatory Bowel Disease Centre, Division of Gastroenterology, Hepatology and Nutrition, The Hospital for Sick Children, Toronto, Ontario, Canada; Child Health Evaluative Sciences, SickKids Research Institute, The Hospital for Sick Children, Toronto, Ontario, Canada; ICES, Toronto, Ontario, Canada; Department of Paediatrics, Temerty Faculty of Medicine, University of Toronto, Toronto, Ontario, Canada; Institute of Health Policy, Management and Evaluation, Dalla Lana School of Public Health, University of Toronto, Toronto, Ontario, Canada; SickKids Inflammatory Bowel Disease Centre, Division of Gastroenterology, Hepatology and Nutrition, The Hospital for Sick Children, Toronto, Ontario, Canada; Child Health Evaluative Sciences, SickKids Research Institute, The Hospital for Sick Children, Toronto, Ontario, Canada; Departments of Medicine and Community Health Sciences, University of Calgary, Calgary, Alberta, Canada; Department of Internal Medicine, Max Rady College of Medicine, Rady Faculty of Health Sciences, University of Manitoba, Winnipeg, Manitoba, Canada; University of Manitoba IBD Clinical and Research Centre, Winnipeg, Manitoba, Canada; Division of Gastroenterology and Hepatology, McGill University Health Centre IBD Centre, McGill University, Montréal, Quebec, Canada; Departments of Medicine, Clinical Health, and Epidemiology, Dalhousie University, Halifax, Nova Scotia, Canada; Crohn’s and Colitis Canada, Toronto, Ontario, Canada; Department of Medicine, University of Ottawa, Ottawa, Ontario, Canada; The Ottawa Hospital IBD Centre, Ottawa, Ontario, Canada; Division of Gastroenterology and Hepatology, Mount Sinai Hospital, University of Toronto, Toronto, Ontario, Canada; Department of Community Health and Epidemiology, University of Saskatchewan, Saskatoon, Saskatchewan, Canada; College of Nursing, University of Saskatchewan, Saskatoon, Saskatchewan, Canada; Crohn’s and Colitis Canada, Toronto, Ontario, Canada; SickKids Inflammatory Bowel Disease Centre, Division of Gastroenterology, Hepatology and Nutrition, The Hospital for Sick Children, Toronto, Ontario, Canada; Child Health Evaluative Sciences, SickKids Research Institute, The Hospital for Sick Children, Toronto, Ontario, Canada; SickKids Inflammatory Bowel Disease Centre, Division of Gastroenterology, Hepatology and Nutrition, The Hospital for Sick Children, Toronto, Ontario, Canada; Child Health Evaluative Sciences, SickKids Research Institute, The Hospital for Sick Children, Toronto, Ontario, Canada; SickKids Inflammatory Bowel Disease Centre, Division of Gastroenterology, Hepatology and Nutrition, The Hospital for Sick Children, Toronto, Ontario, Canada; Child Health Evaluative Sciences, SickKids Research Institute, The Hospital for Sick Children, Toronto, Ontario, Canada; Departments of Medicine and Community Health Sciences, University of Calgary, Calgary, Alberta, Canada; Departments of Medicine and Community Health Sciences, University of Calgary, Calgary, Alberta, Canada; Crohn’s and Colitis Canada, Toronto, Ontario, Canada; Departments of Medicine and Community Health Sciences, University of Calgary, Calgary, Alberta, Canada; École de kinésiologie et des sciences de l’activité physique, Faculté de médecine, Université de Montréal, Montreal, Quebec, Canada; Centre de recherche de l’Institut universitaire de gériatrie de Montréal, Montreal, Quebec, Canada; Crohn’s and Colitis Canada, Toronto, Ontario, Canada; Crohn’s and Colitis Canada, Toronto, Ontario, Canada; James Smith Cree Nation, Saskatchewan, Canada; Muskoday First Nation, Saskatchewan, Canada; Departments of Medicine and Community Health Sciences, University of Calgary, Calgary, Alberta, Canada

**Keywords:** Crohn’s disease, Epidemiology trends, Incidence, Prevalence, Ulcerative colitis

## Abstract

Inflammatory bowel disease (IBD), consisting of Crohn’s disease and ulcerative colitis, is recognized across the world, though Canada has among the highest burdens of IBD in the world. The Canadian Gastro-Intestinal Epidemiology Consortium (CanGIEC) led a six-province study that demonstrated the compounding prevalence of IBD in Canada from 400 per 100,000 in 2002 to 636 per 100,000 in 2014. The prevalence in 2023 is estimated at 825 per 100,000, meaning that over 320,000 people in Canada are living with IBD. Prevalence is forecasted to rise by 2.44% per year such that 1.1% of the population, 470,000 Canadians, will live with IBD by 2035. The overall incidence of IBD in 2023 is 30 per 100,000 person-years, indicating that over 11,000 Canadians will be newly diagnosed with IBD in 2023. Incidence is forecasted to rise by 0.58% per year up to 32.1 per 100,000 by 2035. The rising incidence of IBD is propelled by pediatric-onset IBD, which is rising by 1.23% per year from 15.6 per 100,000 in 2023 to 18.0 per 100,000 in 2035. In contrast, incidence rates among adults and seniors are relatively stable. Understanding the determinates of IBD has expanded through prospective cohort studies such as the Crohn’s and Colitis Canada Genetic, Environmental, Microbial (CCC-GEM) project. Consensus recommendations towards diet, lifestyle, behavioural and environmental modifications have been proposed by international organizations with the goal of optimizing disease control and ultimately preventing the development of IBD. Despite these efforts, Canadian healthcare systems will need to prepare for the rising number of people living with IBD.

**Key Points**

1. IBD evolves across four epidemiologic stages: Emergence (Stage 1), Acceleration in Incidence (Stage 2), Compounding Prevalence (Stage 3), and Prevalence Equilibrium (Stage 4). Canada is currently rooted in the third epidemiologic stage (Compounding Prevalence) where the prevalence of IBD is steadily climbing due to incidence greatly outpacing mortality.
2. Across all provinces, the prevalence of IBD is significantly increasing. In 2023, it is estimated that the prevalence across the provinces ranges from 720 per 100,000 (95% CI: 688, 751) in Manitoba to 968 per 100,000 (95% CI: 879, 1056) in Alberta. Contrarily, incidence across provinces is heterogeneous. It is significantly increasing in BC and QC, stable in AB and ON, and significantly decreasing in MB and SK.
3. In 2023, approximately 322,600 (0.82%, or 8.2 per 1,000) Canadians are estimated to live with IBD. By 2035, that number is expected to rise to 470,000 Canadians (1.1% or 1 in 91), similar to trends reported in the 2018 Impact of IBD in Canada.
4. In 2023, the incidence of IBD is 30 per 100,000 person-years, meaning that over 11,000 Canadians were newly diagnosed with IBD in 2023.
5. Overall, the incidence of IBD is rising by 0.58% per year. Pediatric-onset IBD is the predominant driver of rising incidence at 1.23% per year with the incidence remaining stable among adults and seniors with IBD.
6. If trends in incidence remain the same over the next decade, then incidence will climb to 32.1 per 100,000 in 2035; representing 14,000 Canadians newly diagnosed with IBD in 2035.
7. Information from cohort studies like the Crohn’s and Colitis Canada Genetic, Environmental, Microbial project offers potential strategies for guiding diet, lifestyle, behavioural, and environmental modifications to improve the care of IBD.
8. The incidence of IBD in Canada is recognized across race and ethnicity including Indigenous populations and those of South Asian descent.
9. Canadian healthcare systems will need to contend with the ongoing rising burden of IBD.

## SUMMARY OF CROHN’S AND COLITIS CANADA’S 2018 IMPACT OF IBD: EPIDEMIOLOGY

Crohn’s and Colitis Canada’s 2018 Impact of IBD report on the epidemiology of IBD documented that Canada had among the highest burdens of IBD in the world. In 2018, the prevalence of IBD in Canada was roughly 0.7% of the population, which represented approximately 270,000 Canadians living with IBD, with half living with Crohn’s disease. Forecasting projected a steadily rising prevalence towards 1% of the Canadian population over the next decade. By 2030, models estimated that nearly 400,000 Canadians would be living with IBD. The highest incidence was reported in Nova Scotia and the lowest in British Columbia. While IBD can be diagnosed at any age, adolescents and young adults had the highest incidence of the disease. In the past, Caucasians of European descent and Ashkenazi Jews were thought to be at the highest risk for IBD. However, IBD is now recognized among all races and ethnicities; in particular, among the first-generation children of immigrants to Canada. Consequently, healthcare systems need to prepare for the rising burden of IBD in Canada.

## INTRODUCTION

Inflammatory bowel disease (IBD), consisting of Crohn’s disease and ulcerative colitis, are chronic inflammatory disorders of the intestines. IBD is believed to arise among genetically susceptible individuals who are exposed to environmental exposures that alter their intestinal microbiome.^1^ Over 200 genes have been identified that are associated with altering the risk of developing IBD.^2^ Most of these genes are involved with the intestinal immune system’s interaction with the microbiome.^3^ Environmental exposures, particularly those early in life, that influence the composition and diversity of the microbiome may increase the risk of developing IBD later in life.^4^

The environmental determinants of IBD are rooted in the westernization and industrialization of society.^5^ The incidence of IBD escalated during the latter half of the 20th century in the early-industrialized world: North America, Western Europe, and Oceania.^6, 7^ At the turn of the 21st century, the incidence of IBD has levelled in some regions of the early-industrialized world; however, it has begun to rise dramatically in newly industrialized countries in Asia and Latin America as these countries have begun to undergo westernization and urbanization.^8^ Today, IBD is recognized as a global disease.

In countries like Canada, the prevalence of IBD has steadily been rising.^9^ Decades of rising incidence in conjunction with low mortality means that individuals with IBD are continually added to gastroenterology clinics, whereas few leave.^10^ Consequently, healthcare systems must contend with the ever-expanding volume of individuals living with IBD.^11^

The purpose of this article is to understand the evolution of IBD across epidemiologic stages, highlight the most recent data on the incidence and prevalence of IBD in Canada, and explore how the determinants of IBD may be modified to reduce the incidence of IBD in Canada.

## THE FOUR EPIDEMIOLOGIC STAGES IN THE EVOLUTION OF IBD ACROSS THE WORLD

**Figure 1.**
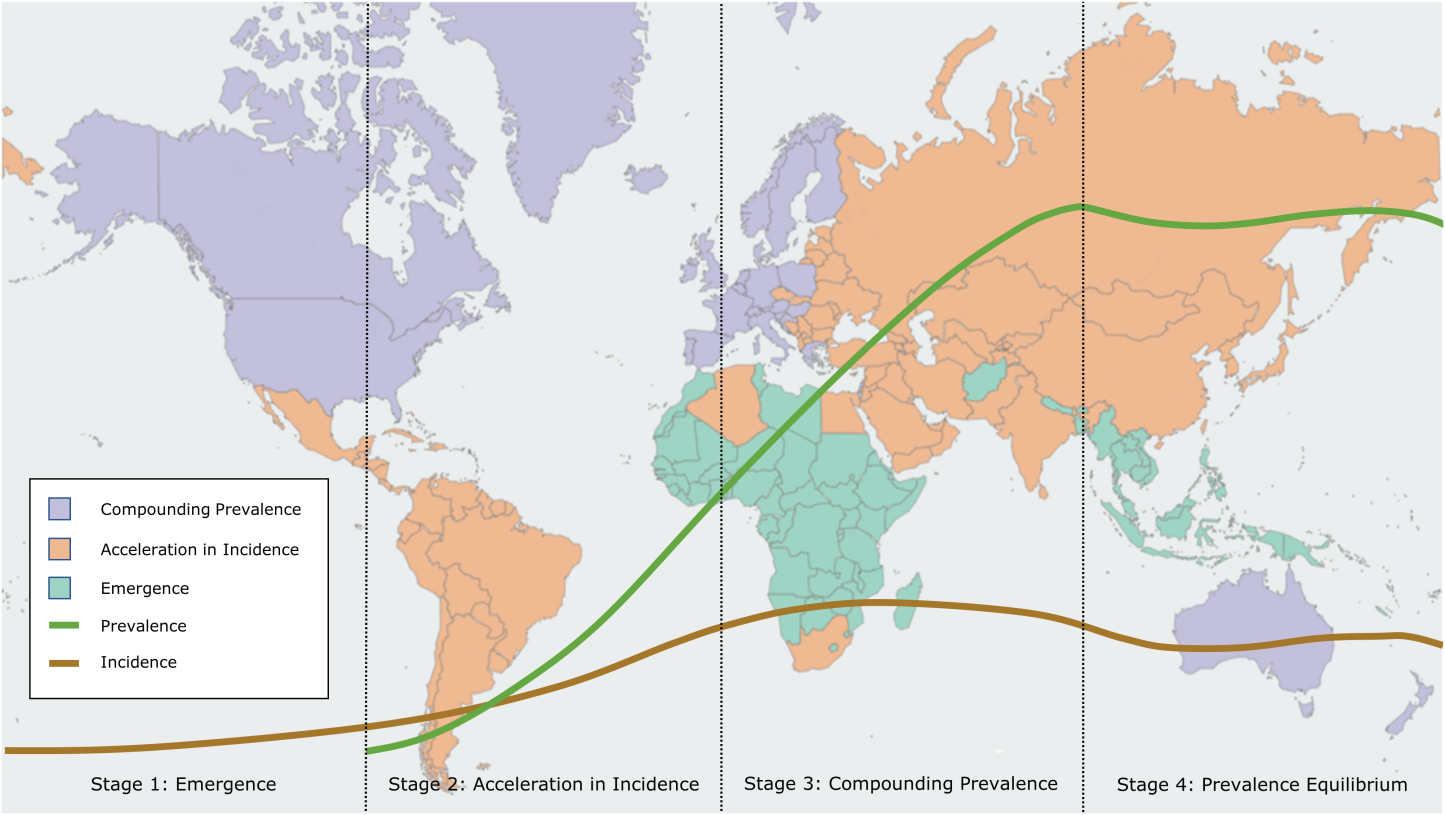
This figure depicts a stylized track of incidence and prevalence rates in each of the four epidemiologic stages. In Stage 1, Emergence, there is very low incidence due to sporadic disease. In Stage 2, Acceleration in Incidence, incidence rates quickly increase, and prevalence starts to accumulate. In Stage 3, Compounding Prevalence, incidence stabilizes but prevalence continues to climb. In Stage 4, Prevalence Equilibrium, the prevalence of disease begins to stabilize due to stable incidence and an aging population. Every region of the world is currently in one of the first three stages, as depicted by colours in the background map. No region has yet transitioned to Stage 4. (Adapted from Kaplan & Windsor 2021,^10^ updated with data from Windsor et al. 2023.^13^)

A theoretical framework for the global evolution of IBD across four epidemiologic stages has been proposed (Figure 1).^10^ The first epidemiologic stage, Emergence, postulates that in developing countries, IBD arises as sporadic, incident cases in the population. In this stage, both the incidence and the prevalence of IBD are very low. With economic expansion, newly industrialized countries experience advances in healthcare infrastructure and westernization of lifestyle and diet. These societal changes trigger the escalation in the incidence of IBD, consistent with the second epidemiologic stage, Acceleration in Incidence. During the second epidemiologic stage, the incidence rate rises sharply, whereas the prevalence of IBD remains low in the population. IBD is predominately diagnosed in young individuals and has low mortality. Thus, new cases are continually added to the prevalent base with few dying. As long as incidence exceeds mortality, prevalence will steadily rise, leading to the third epidemiologic stage, Compounding Prevalence. During the third epidemiologic stage, the previously sharp rising slope of incidence decelerates; the incidence rate may stabilize or even decline in some regions. However, the slope of prevalence increases continues to rise steadily. The fourth epidemiologic stage, Prevalence Equilibrium, is a theoretical stage wherein prevalence begins to stabilize as a result of rising mortality from an aging IBD population.^10^

Understanding how societies transition across the different epidemiologic stages allows us to historically evaluate epidemiologic patterns, estimate the current incidence and prevalence of IBD, and forecast temporal changes in the burden of IBD into the future.^10^ Canada was firmly rooted in the second epidemiologic stage during the latter half of the 20th century with steadily rising incidence and relatively low prevalence of IBD.^12^ At the turn of the 21st century, the acceleration in incidence began to slow down in Canada; Canada is now in the compounding prevalence stage of the epidemiologic evolution of IBD.^9^

## THE PREVALENCE OF IBD IS STEADILY CLIMBING IN CANADA ACROSS ALL AGE GROUPS

The Canadian Gastrointestinal Epidemiology Consortium (CanGIEC) is a national network of IBD epidemiologists in Canada who analyzed provincial surveillance cohorts of IBD from six provinces (British Columbia, Alberta, Saskatchewan, Manitoba, Ontario, and Quebec) representing over 95% of the Canadian population; this is updated data, separate from a prior forecasting analysis undertaken by the same group.^9, 14^ Each of the provinces has data from 2002 to 2014, but data from Manitoba begins as early as 1987, and data from British Columbia extends to 2019. Overlapping years from the six provinces were used to create a national model of prevalence in Canada, to derive the average annual percent change (AAPC), and to forecast prevalence to 2035.^14^

The prevalence of IBD steadily increased from 2002 (389 per 100,000) to 2014 (636 per 100,000; 321 per 100,000 for Crohn’s disease, and 315 per 100,000 for ulcerative colitis and IBD-unclassified [IBD-u] together). After 2014, the national prevalence of IBD is forecasted to rise by an AAPC of 2.44% per year. The rising prevalence over time is similar for both Crohn’s disease and ulcerative colitis. Overall, the prevalence of IBD in 2023 is estimated to be 825 per 100,000 (410 per 100,000 for Crohn’s disease, and 414 per 100,000 for ulcerative colitis and IBD-u). A 0.82% prevalence represents 322,600 people living with IBD in Canada. By 2035, the prevalence is forecasted to climb to 1.08% of the population, representing 470,000 Canadians living with IBD. Prevalence across all age strata was forecast to significantly increase. The highest AAPC was seen in the elderly, with a prevalence of 841 per 100,000 in 2014 and 1534 per 100,000 in 2035.

Few studies have been done specifically to identify IBD within an Indigenous population in Canada—the main one coming from Saskatchewan.^15^ Similar to other prevalence studies in Canada,^16^ the prevalence of IBD in Indigenous populations is significantly increasing at 4.2% per year (95% CI: 3.2, 5.2), which is an increase from 64 per 100,000 (95% CI: 62, 66) in 1999 to 142 per 100,000 (95% CI: 140, 144) in 2016.^15^

The prevalence of IBD in Canada is analogous to other countries of the early-industrialized world including, the United States, Western Europe, and Oceania, which have reported similar trends in compounding prevalence.^8^ For example, forecast models in Scotland suggest that the prevalence of IBD was 0.78% in 2018 and will approximate 1% of the population by 2028.^17^

## THE INCIDENCE OF IBD IS RISING IN CANADA, PREDOMINANTLY DRIVEN BY CHILDREN WITH IBD

CanGIEC explored the incidence of IBD provincially (BC, AB, SK, MN, ON, and QC) and in a national model across 2007–2014. Historical data derived from provincial administrative healthcare databases were used to forecast incidence trends into the future.

In 2014, the calculated incidence of IBD across Canada was 28.4 per 100,000 person-years (13 per 100,000 for Crohn’s disease, and 15.4 per 100,000 for ulcerative colitis and IBD-u). After 2014, the national incidence of IBD is forecast to rise by an AAPC of 0.58%. The rise in incidence in Canada is predominantly driven by ulcerative colitis and IBD-u, which are projected to increase by 1.23% per year. In contrast, the incidence of Crohn’s disease is forecast to remain stable. Overall, the incidence of IBD in Canada is estimated to be 30 per 100,000 person-years in 2023, with an incidence of 12.2 per 100,000 for Crohn’s disease and 17.5 per 100,000 for ulcerative colitis and IBD-u. These values represent over 11,700 Canadians being newly diagnosed with IBD in the year 2023—approximately 4800 with Crohn’s disease and 6800 with ulcerative colitis or IBD-u. Moreover, with the projected rise in the incidence of 0.58% per year, the incidence of IBD is forecast to be 32.1 per 100,000 in 2035 (11.5 per 100,000 for Crohn’s disease, 20.1 per 100,000 for ulcerative colitis and IBD-u). Consequently, if trends in incidence remain the same over the next decade, then 14,000 Canadians will be newly diagnosed with IBD in 2035.

The incidence of pediatric-onset IBD was 13.9 per 100,000 in 2014 and is forecast to rise by 1.23% per year up to 18.0 per 100,000 in 2035. In contrast, the incidence of IBD diagnosed in adults and seniors over the age of 64 years has been stable and is forecast to remain stable over the next decade. The incidence of IBD is similar between the sexes: 28.9 per 100,000 in females and 30.0 per 100,000 in males in 2023.

Analyzing provinces separately yields varying trends in incidence, in part due to differences in data availability across years. The incidence rates of IBD were stable in Alberta (2007–2017, AAPC: −0.75; 95% CI: −2.29, 0.36) and Ontario (1999–2016, AAPC: 0.83; 95% CI: −1.55, 2.29), increasing in British Columbia (1999–2019, AAPC: 0.71; 95% CI: 0.45, 0.95) and Quebec (2002–2014, AAPC: 0.58; 95% CI: 0.15, 0.95), and decreasing in Manitoba (1987–2014, AAPC: −0.93; 95% CI: −1.51, −0.47) and Saskatchewan (1998–2018 AAPC: −7.72; 95% CI: −21.58, −2.56).^14^ Future studies are necessary to explain the heterogeneity in temporal incidence trends between provinces.

Historically, the incidence of IBD has been the highest among Caucasians living in Canada. However, at the turn of the 21st Century, IBD is recognized across races and ethnicities including children of immigrants from newly industrialized countries.^18^ For example, there was a similar incidence among those of South Asian ethnicity living in Canada compared to the general population.^19^ Furthermore, incidence among Indigenous populations in Saskatchewan has remained stable (AAPC: −2.7%; 95%CI: −6.2, 0.8) with a rate of 11 per 100,000 (95% CI: 5, 25) in 1999 and 3 per 100,000 (95% CI: 1, 11) in 2016. Additionally, a higher rate of ulcerative colitis than Crohn’s disease among the Indigenous population in this study was observed (ulcerative colitis to Crohn’s disease ratio of 1.71) while population-based Canadian studies show equal rates of ulcerative colitis and Crohn’s disease.^15, 16^ Future epidemiological studies should evaluate the evolving heterogeneity of IBD across race and ethnicity.

## MODIFYING ENVIRONMENTAL RISK FACTORS MAY PREVENT IBD AND HELP CONTROL DISEASE ACTIVITY

If historical trends continue, the prevalence of IBD in Canada will steadily rise over the next decade to levels that are significantly higher than what we currently see. However, the rising prevalence can be slowed down by interventions aimed to reduce incidence. Prevention of IBD is predicated on learning the underlying determinates that drive the pathogenesis of IBD and modifying those factors that influence the development of IBD. Over the past 5 years, tremendous insight into the environmental determinants of IBD has been discovered.

Several prospective cohort studies on the environmental risk factors of IBD offer indirect evidence on potential preventative strategies towards IBD and control of disease activity. The International Organization for the study of Inflammatory Bowel Disease (IOIBD) published dietary guidance for those with IBD.^20^ Whole foods including vegetables, fruits, and omega-3 oils from fish are associated with a reduced risk of developing IBD and sustaining remission in those with IBD.^20, 21^ In contrast, foods that should be minimized in those with IBD include saturated and trans fats, red and processed meats, and refined sugars. Moreover, highly processed foods, emulsifiers, and artificial sweeteners may worsen the risk of IBD.^20-22^ Most dietary effects are believed to be driven by alterations in the composition and diversity of the intestinal microbiome.^20^ However, recent Canadian data suggest certain types of fibres may trigger inflammation in some individuals, further complicating our understanding of diet’s role in the risk and prognosis of IBD.^23^

IOIBD has also published consensus recommendations towards lifestyle, behavioural, and environmental modifications geared towards care for those with IBD.^24^ The key recommendations from that report included avoiding smoking, eating a healthy whole-food diet such as the Mediterranean diet, minimizing regular use of high-dose NSAIDs, targeting a normal BMI, engaging in regular physical activity, and promoting mental healthcare.^24^ Recommendations for the children of those with IBD who are at higher risk of acquiring IBD and population-level interventions geared towards IBD prevention have been considered. Based on indirect observational research, guidance includes: Never start smoking; minimize NSAIDs in adulthood and antibiotics in childhood; ensure adequate vitamin D; optimize fruits, vegetables, fibre, and fish oils in diet; promote physical activity, regular sleep patterns, stress reduction, and a healthy weight; and breastfeeding where possible.^24^

The lack of randomized controlled interventional studies and high-quality, population-level environmental modification studies is a limitation of current guidelines. Future studies based on translational and clinical research that supports the design of high-quality, environmental interventions that demonstrate IBD prevention strategies are necessary to reduce the future incidence of IBD.

## THE CCC-GEM PROJECT IS SHINING LIGHT ON THE DETERMINATES OF IBD

The Crohn’s and Colitis Canada Genetic, Environmental, Microbial (CCC-GEM) project is a prospective cohort study that has followed over 5000 first-degree relatives of individuals with Crohn’s disease. Since CCC-GEM’s inception in 2008, over 120 new diagnoses of Crohn’s disease or ulcerative colitis have been made within the cohort. Analyses from the CCC-GEM cohort suggest that Crohn’s disease may be identified prior to a clinical diagnosis. Antibodies to microbes^25^ and alterations in the intestinal permeability^26^ were associated with the future development of Crohn’s disease.

Identifying individuals prior to a clinical diagnosis of IBD opens the possibility of intervening through environmental modifications with the goal of preventing disease development.

For example, the subset of the CCC-GEM cohort that followed a Mediterranean diet underwent changes in the microbiome that were associated with reduced intestinal inflammation before the diagnosis of Crohn’s disease.^27^ Studies such as CCC-GEM help to bridge the knowledge gap around environmental determinants and their modification by allowing for the identification of possible environmental factors and their effects in a real-world setting. Through the monitoring of thousands of individuals, this study works to identify the environmental determinants, along with interacting genetics, aiming to decrease the incidence of Crohn’s disease. This decrease in incidence would in turn decrease the prevalence of the disease and provide a necessary decrease in burden.

## CONCLUSION

Crohn’s disease and ulcerative colitis are global diseases, with Canada reporting among the highest prevalence and incidence of IBD in the world. In Canada, the prevalence of IBD climbed from 400 per 100,000 in 2002 to 636 per 100,000 in 2014. Today, in 2023, the prevalence is estimated to be 825 per 100,000 (approximately 0.8% of the population) and is forecast to rise by 2.44% per year over the next decade. In 2023, over 320,000 people in Canada are living with IBD, with prevalence forecasted to be almost 1.1% of the population by 2035, representing 470,000 Canadians with IBD. The overall incidence of IBD in Canada in 2023 is 30 per 100,000, meaning 11,000 new diagnoses of IBD were made in 2023. Incidence is projected to rise by 0.58% per year, reaching 32.1 per 100,000 by 2035. The rise in incidence is predominately driven by pediatric-onset IBD with relatively stable rates among adults and seniors. Addressing the rising burden of IBD in Canada will require a concerted effort to understand the underlying determinants of IBD and to use this information to prevent disease development.

## KNOWLEDGE GAP AND FUTURE RESEARCH DIRECTIONS

1. The reason for the rising incidence of IBD in children and adolescents, with stable rates in adults and seniors, is not known. Future research should explore environmental exposures driving the rising incidence of pediatric-onset IBD.
2. Temporal trends in the incidence of IBD varies by province. Future studies should explore the heterogeneity of incidence rates over time between provinces.
3. Intervention studies on modifiable diet, lifestyle, behavioural, and environmental factors that reduce the incidence of IBD are necessary.
4. Population-level guidelines directed by high-quality, intervention-based research are necessary to support activities that prevent IBD development.
5. The burden of IBD among Indigenous populations and the first-generation children of immigrants are on the rise, but there is a paucity of research in these areas; this should be an area of focus for future research and for changes in health policy to ensure that we accommodate the needs of these populations.

## PATIENT AND CAREGIVER PERSPECTIVE

Patient partners highlighted the importance of monitoring the IBD prevalence and incidence trends in Canada, especially the rising numbers observed among children. The epidemiology of IBD among specific populations (e.g., Indigenous peoples and first-generation immigrants) also requires the attention of different stakeholders and better awareness of healthcare providers about these numbers and their implications. Based on this epidemiology, the IBD community can inform how healthcare systems could be well-equipped (e.g., outpatient care and after-hours services to avoid visits to the emergency room) and advocate for providing adequate health services. Individuals with IBD can contribute crucial information to help prepare Canadian healthcare systems for reforming and designing efficient and timely IBD care. In addition, patient partners underlined the importance of the risk factors for IBD to, potentially, modify them and diagnose IBD before its clinical onset. If we focus on prevention and early diagnosis of IBD, the growing burden of IBD on the healthcare systems could be diminished, as well as the burden of this chronic condition on the well-being of individuals living with IBD in Canada.

## POLICY IMPLICATIONS AND KEY ADVOCACY OUTCOMES

1. Due to the increasing prevalence of IBD in Canada, the Canadian healthcare systems need to prepare for the increasing burden (e.g., more gastroenterologists) to ensure afflicted individuals receive high-quality care as needed to manage long-standing diseases or flares.
2. Under-represented populations—such as Indigenous populations in Canada and first-generation children of immigrants to Canada—require more research to understand the incidence and prevalence of IBD therein. Current research is lacking, and data need to be made available in order to better grasp the burden that is imparted on these populations. It is critically important to engage in knowledge translation with these stakeholder groups and to make healthcare providers aware of this information to challenge biases and knowledge gaps.
3. Increased funding needs to be geared towards research that looks at the development of IBD and possible ways to mitigate its development.
4. Cross-organizational partnership is encouraged between Crohn’s and Colitis Canada and other agencies to promote healthy lifestyle choices, particularly around diet and other modifiable behaviours that impact the risk of developing IBD.

## SUPPLEMENT SPONSORSHIP

This article appears as part of the supplement “The Impact of Inflammatory Bowel Disease in Canada in 2023”, sponsored by Crohn’s and Colitis Canada, and supported by Canadian Institutes of Health Research Project Scheme Operating Grant (Reference number PJT-162393).

## Data Availability

No new data were generated or analyzed in support of this review.
