# Correction to: The 2023 Impact of Inflammatory Bowel Disease in Canada: Epidemiology of IBD

**DOI:** 10.1093/jcag/gwad043

**Published:** 2023-11-01

This is a correction to: Stephanie Coward, Eric I Benchimol, M Ellen Kuenzig, Joseph W Windsor, Charles N Bernstein, Alain Bitton, Jennifer L Jones, Kate Lee, Sanjay K Murthy, Laura E Targownik, Juan-Nicolás Peña-Sánchez, Noelle Rohatinsky, Sara Ghandeharian, James H B Im, Tal Davis, Jake Weinstein, Quinn Goddard, Julia Gorospe, Jennifer Bennett, Léa Caplan, Maxime Bergevin, Xin Yu Yang, Kate Mason, Rhonda Sanderson, Colten Brass, Gilaad G Kaplan, The 2023 Impact of Inflammatory Bowel Disease in Canada: Epidemiology of IBD, Journal of the Canadian Association of Gastroenterology, Volume 6, Issue Supplement_2, September 2023, Pages S9–S15, https://doi.org/10.1093/jcag/gwad004

In the originally published version of this manuscript, author Julia Gorospe was inadvertently omitted from the author list.

The ‘Key Points’ section contained an error, whereby ‘1000,000’ should have been given as ‘100,000’ in Point 6:

“If trends in incidence remain the same over the next decade, then incidence will climb to 32.1 per 1000,000 in 2035; representing 14,000 Canadians newly diagnosed with IBD in 2035.”

The manuscript has been corrected accordingly.

